# Protein Intake Mediates the Relationship of Sleep Quality with Handgrip Strength in Older Persons 60+ Years

**DOI:** 10.1093/geroni/igaf122.2437

**Published:** 2025-12-31

**Authors:** Marie Fanelli Kuczmarski, Elizabeth Orsega-Smith, May Beydoun, Michele Evans, Alan Zonderman

**Affiliations:** University of Delaware, Newark, Delaware, United States; University of Delaware, Newark, Delaware, United States; National Institute on Aging, Baltimore, Maryland, United States; National Institute on Aging, Baltimore, Maryland, United States; National Institute on Aging, Baltimore, Maryland, United States

## Abstract

Handgrip strength, a measure of muscle strength and biomarker for health and vitality, may be influenced by diet, physical activity, and sleep. The study objective was to determine if protein intake/body weight (gm/kg) and engagement in physical activity mediated the relationship between sleep with handgrip strength in persons ≥ 60 years examined in the HANDLS study, 2013-2017. Sleep quality was measured using the Pittsburgh Sleep Quality Index questionnaire. Handgrip strength was measured with Jamar Hydraulic Hand Dynamometer and expressed per Body Mass Index (based on measured height and weight). Protein intake was calculated from 2-24-hour recalls collected with the USDA Automated Multiple-Pass Method. Physical activity was self-reported and expressed as meeting the Life Simple 7 criterion (≥150 mins/week, 0-149 mins/week, 0 mins/wk). Mediation analysis used Hayes PROCESS macro, model #4, for SPSS Version 4.2. Before performing the analysis,138 individuals with sleep apnea were excluded, resulting in an analytical sample of 1308. Covariates that were adjusted for in models included: age (years), sex at birth (male, female), self-reported race (African American, White), poverty status (< 125% or > 125% U.S. HHS Poverty Guidelines), current cigarette smoker (yes, no), marijuana, opiate and/or cocaine user (yes, no), medical conditions of diabetes, hypertension, and/or metabolic syndrome (yes, no), and mean energy(kcal). Protein intake, but not physical activity, significantly influenced handgrip strength (p = 0.002) and mediated the relationship of sleep quality with handgrip (R2 = 0.47). Results suggest that protein is critical to preserving muscle strength and plays a role in sleep quality.

